# Evaluation of the Infectious Potential of *Neoparamoeba perurans* Following Freshwater Bathing Treatments

**DOI:** 10.3390/microorganisms9050967

**Published:** 2021-04-29

**Authors:** Richard S. Taylor, Joel Slinger, Chris Stratford, Megan Rigby, James W. Wynne

**Affiliations:** 1 CSIRO Agriculture and Food, Castray Esplanade, Hobart, TAS 7000, Australia; megan.rigby@csiro.au (M.R.); james.wynne@csiro.au (J.W.W.); 2 CSIRO Agriculture and Food, Bribie Island Research Centre, Woorim, QLD 4507, Australia; joel.slinger@csiro.au (J.S.); chris.stratford@csiro.au (C.S.); 3 Institute for Marine and Antarctic Studies, University of Tasmania, Launceston, TAS 7250, Australia

**Keywords:** amoebic gill disease (AGD), Atlantic salmon, *Salmo salar*, integrated pest management, salinity, freshwater tolerance, wellboat

## Abstract

Freshwater bathing for 2–3 h is the main treatment to control amoebic gill disease of marine-farmed Atlantic salmon. Recent in vitro studies have demonstrated that amoebae (*Neoparamoeba perurans*) detach when exposed to freshwater and that some eventually reattach to culture plates when returned to seawater. Here, we evaluated the potential for gill-detached *N. perurans* to survive a commercially relevant treatment and infect AGD-naïve fish and whether holding used bathwater for up to 6 h post treatment would lower infectivity. AGD-affected fish were bathed in freshwater for 2 h. Naïve salmon were exposed to aliquots of the used bathwater after 2, 4, 6 and 8 h. The inoculation was performed at 30 ppt for 2 h, followed by gradual dilution with seawater. Sampling at 20 days post inoculation (dpi) and 40 dpi confirmed rapid AGD development in fish inoculated in 2 h used bathwater, but a slower AGD development following exposure to 4 h bathwater. AGD signs were variable and reduced following longer bathwater holding times. These results suggest that viable amoebae are likely returned to seawater following commercial freshwater treatments, but that the risk of infection can be reduced by retention of bathwater before release.

## 1. Introduction

Parasites are ubiquitous in the aquatic environment, but in the wild their impact on fish health is generally kept in balance by host–parasite interactions, including parasite lifecycle, host age, immune function, and behavior. Where fish are exposed to biotic or abiotic stressors, their susceptibility to parasitic disease can increase [1,2,3]. Fish reared in intensive sea pen monoculture are not co-evolved with local parasite populations and are particularly susceptible to parasitic diseases due to high stocking density, in-pen environment and handling stressors [4]. However, sea pen aquaculture generally favors ectoparasites and a reduction in parasite diversity because trophic transmission of parasites with complex lifecycles is generally reduced through limited access to wild prey and the use of manufactured feeds [5]. Parasites with single host lifecycles are most likely to proliferate in sea pens because they may reproduce rapidly and can directly reinfect farmed fish hosts or spread to nearby farms [6,7]. The economic impacts of parasitism to intensive aquaculture accrue directly through mortality, reduced growth, poor feed conversion and harvest quality, increased disease susceptibility of stressed fish and the costs and effects of husbandry and management practices, such as monitoring, treatment, fallowing, grading, and stock handling.

Control of ectoparasites in intensive aquaculture has primarily taken a treatment-based approach, with the aim of reducing parasite load and host pathology to minimize the adverse impact of disease on fish welfare and production. However, experience from terrestrial agriculture has repeatedly demonstrated that parasites can rapidly develop resistance to homogenous treatment regimes, leading to decreased efficacy [8]. In Atlantic salmon (*Salmo salar*) aquaculture, caligid copepods (*Lepeoptheirus salmonis* and *Caligus* sp.) have adapted resistance to a range of chemical treatments [9,10,11]. There has been increased focus on non-medicinal treatment methods including exposure of salmon to freshwater [12,13]. However, recent evidence suggests that *L. salmonis* life stages detached from fish during freshwater treatment may survive and reattach when returned to seawater [14,15]. With increased focus on integrated pest management approaches to break parasite lifecycles through fallowing, year class separation and killing or avoiding infective stages, recent advances in wellboat design include 100 µm filtration of used treatment freshwater to prevent the release of viable freshwater resistant sea lice back into the marine environment [14].

Freshwater bathing has been the main treatment for amoebic gill disease (AGD) in Tasmania (Australia) since the late 1980s [16] and has been increasingly adopted to treat AGD in Ireland, Scotland and Norway over the past decade [12]. Caused by the amphizoic marine amoeba *Neoparamoeba perurans* [17,18], attachment of amoeba trophozoites to salmon gills triggers a localized tissue response including epithelial hyperplasia, hypertrophy and lamellar fusion and is grossly typified by raised white mucoid lesions [19]. If untreated, detrimental gill pathology increases in affected farm cohorts, leading to reduced productivity and increased mortality [20,21]. 

Freshwater AGD treatment was originally applied by holding affected fish in a freshwater-filled tarpaulin liner for 2–3 h and then removing the tarpaulin to release the fish back into the pen while dispersing the used freshwater. Early research [22] showed that treatment became less effective with low levels of saltwater contamination (between 2.2 and 4.1 ppt). Although farmers aim to treat at 0–1 ppt, treatment time is extended above 2 ppt and an upper threshold of 3 ppt is generally accepted [23] before a treatment is cancelled. Treatments are now mostly performed in wellboats which allow close control of water quality and offer the ability to reuse freshwater. While freshwater is effective at reducing clinical signs of AGD, previous research has demonstrated that viable amoebae can remain on the gills post treatment and rapidly proliferate thereafter [24,25,26]. Survival of amoebae on the gills is likely because they are protected by the gill structure and host mucus. Infection also occurs from trophozoites in the farm environment, which can be readily detected in the water column [27,28] and on biofouling, biofilm, sediment and wild fish [29,30]. The reiterative cycle of AGD bathing and reinfection by *N. perurans* is a major economic impact to farm production costs [31].

Over many years of liner-based freshwater treatment, the disposal of used AGD bathwater on farm sites was not considered to be a significant risk of reinfection, because it was understood that freshwater killed the majority of neoparamoebae [32]. However, in vitro studies have shown that *N. perurans* cells acutely exposed to freshwater will rapidly detach from the culture plate to form rounded pseudocysts [32,33,34] and that, following return to seawater conditions, many of these pseudocysts gradually return to amoeboid form and reattach to surfaces. Lima et al. [33] reported that amoeba survival in vitro is inversely related to freshwater exposure time, with survival after 30 min, 60 min, 2 h, 3 h and 4 h of 87.3%, 77.3%, 16.0%, 4.3% and 0%, respectively. These results differed from Wright et al. [34], who found that pseudocysts do not survive for ≥1 h exposure in deionized water. The discrepancy between the two studies may be due to cultured strain differences or to dissimilar freshwater chemistry, which was not specified in either study. Indeed, Powell and Clark [32] reported that increased water hardness (higher concentrations of Mg^2+^ and Ca^2+^) promoted amoeba survival. The formation of pseudocysts is likely a natural adaptation to brackish conditions, allowing the cell to sink below brackish surface layers or to be transported away by water currents. Amoebae surviving in vitro exposure to fresh or brackish water conditions have been seen to form vacuoles to remove freshwater from the cell before returning to the trophozoite form [33,35].

Despite in vitro evidence of *N. perurans* surviving exposure to freshwater for commercially relevant (2–3 h) periods, there have been no studies to examine whether amoebae detached from the gills during freshwater treatment are able to reinfect salmon when returned to the marine environment. The release of viable parasites post treatment could pose an increased risk of localized infection and increased selection for freshwater tolerance. Accordingly, this study seeks to assess whether naïve fish develop AGD following exposure to used AGD bathwater. Furthermore, we examine whether holding the used AGD bathwater for extended periods before release will lower the risk of infection to nearby fish.

## 2. Materials and Methods

### 2.1. Animal Ethics Statement

All animal procedures were approved by the CSIRO Queensland Animal Ethics Committee (CQAEC permit 2019-09) under guidelines of the Australian Code for the Care and Use of Animals for Scientific Purposes (National Health and Medical Research Council, 2013). Fish were sampled non-destructively following anesthesia in 20 mg/L Aqui-S^®^ (Aqui-S, Lower Hutt, New Zealand) or humanely killed with 100 mg/L Aqui-S for destructive sampling.

### 2.2. AGD Bathwater Exposure Trial

This study took place at the CSIRO Bribie Island Research Centre in Queensland, Australia. The seawater supply is pumped ashore and filtered (~40 µm), ozonated (100 g O_3_.h^−1^), UV treated (80 mJ.cm^2^) and chilled before use.

Prior to allocating fish, calibrated graduations were marked every 20 L inside the experimental tanks to ensure that water volumes could be closely gauged during the exposure phase of the experiment. Seven days prior to the experiment, 225 AGD-naïve Atlantic salmon (966 ± 188g, mean ± SD) were transferred from a 5000 L seawater holding tank and allocated evenly across fifteen tightly lidded 500 L tanks (15 fish per tank) supplied with aerated (95.6 ± 2% oxygen saturation) flow-through 20 μm filtered and UV-treated natural 35 ppt salinity seawater. The seawater temperature was gradually increased from 13.6 to 15.1 °C during the holding period (mean 14.1 ± 0.5 °C). The fish were fed commercial feed pellets (Skretting Optiline) to satiation using Arvo-Tec TD2000 auto-feeders controlled by an Arvo-Tec Wolf Controller (Arvo-Tec Oy, Huutokoski, Finland), with waste feed collected and dried twice daily to monitor daily feed intake. Feed intake averaged 0.74 ± 0.1% body weight/day during this initial holding period. The fish were starved overnight prior to the bathwater exposure experiment.

To obtain AGD-affected fish for freshwater bathing, 49 salmon were added to a constant infection tank (CIT) two weeks before the trial. The CIT is managed to maintain a population of fish that are naturally infected with wild-type *N. perurans* through cohabitation with previously infected fish [36] and is housed in a biosecure room separated from experimental tanks. The CIT fish were allowed to develop AGD, aimed at high average gill score > 3.0. On 22 July 2020, 40 fish from the CIT were bathed in a single freshwater bath for 120 min. The bath was performed in 500 L of dechlorinated domestic freshwater at 13.9 °C with oxygen maintained at 115 ± 9.9% saturation (salinity 0.2 ppt). Carbonate hardness (KH) and general hardness (GH) were measured using a KH and GH test kit (API Aquarium Pharmaceuticals, USA) at 7° dKH and 125 mg/L, respectively.

At the completion of the bath, these fish were removed and anesthetized in 20 mg/L Aqui-S. The fish were weighed (898.6 ± 279.3 g) and AGD severity was estimated as a single score (scale 0 to 5) of gross pathology across all 16 gill surfaces according to Taylor et al. [21]. From 5 fish, all right-side gill arches (8 hemibranch surfaces) were non-destructively swabbed using sterile disposable cotton swabs (Westlab, Ballarat, Australia) which were immediately preserved in 2 mL screw cap tubes containing 1.5 mL RNAlater (Life Technologies, Carlsbad, CA, USA).

Immediately post bath, a 120 L aliquot of used bathwater was removed and coarsely filtered to be used for the initial (2 h) used bathwater treatment. The remaining bathwater was maintained at 14 °C by a chilled heat exchanger and continuously aerated to ensure that any gill-detached amoebae were kept suspended in the water column. Every 2 h thereafter, a 120 L aliquot of retained bathwater was removed and coarsely filtered, forming a further three bathwater treatments to examine the effects of holding water prior to release: 4 h (2 h bathwater held for a further 2 h), 6 h (2 h bathwater + 4 h holding) and 8 h (2 h bathwater + 6 h holding). A negative control treatment (unused freshwater) was also included. 

**Figure 1:**
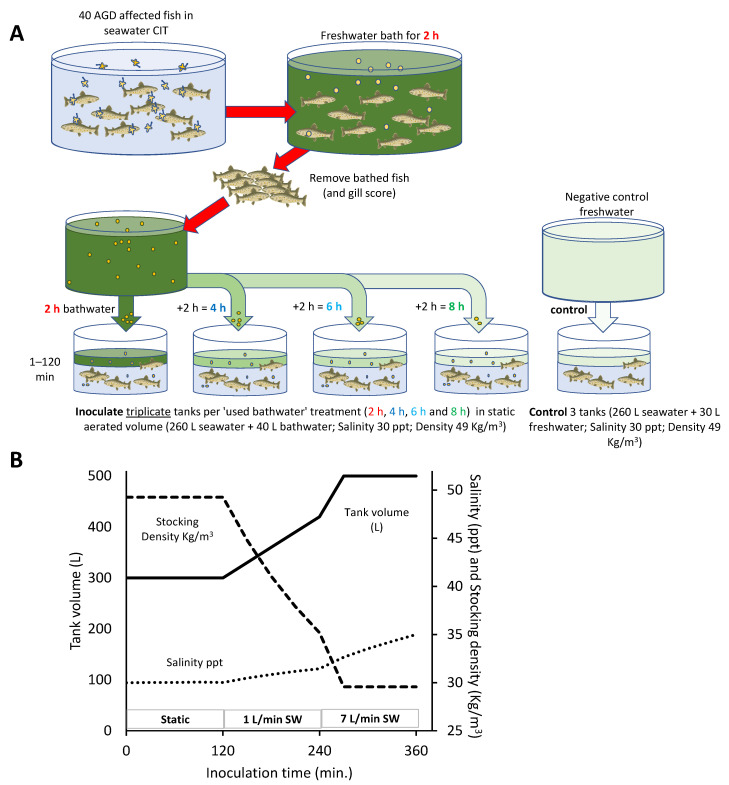
Schematic of the AGD bathwater inoculation process. (**A**) AGD-affected salmon from the constant infection tank (CIT) were bathed in freshwater for 2 h and then removed. Aliquots (40 L) of the used bathwater (“2 h bathwater”) were immediately transferred to triplicate 500 L tanks, each containing 15 naïve salmon in 260 L of seawater, mixing to a combined volume of 300 L and salinity of 30 ppt. The remaining bathwater was retained and further 40 L aliquots transferred out to triplicate inoculation tanks after a further two (“4 h bathwater”), four (“6 h bathwater”) or six hours (“8 h bathwater”). Additionally, triplicate control tanks were exposed to 40 L aliquots of freshwater. (**B**) Illustrative chart of tank volume, fish stocking density and salinity changes during the three inoculation steps. Over the initial 0–120 min step, the fish were held in 300 L of 30 ppt water with aeration and supplementary oxygenation. After 120 min, seawater was introduced at a slow flow (1 L/min) allowing tank volume and salinity to gradually increase to 420 L, 31.5 ppt. At 240 min, the seawater flow was increased to 7 L/min allowing the tank to fill and overflow to the outlet. With continued mixing and displacement with seawater, the salinity gradually increased to 35 ppt.

In order to expose AGD-naïve fish to used AGD bathwater, the water level in each 500 L tank of fish was gradually lowered to 260 L, then 40 L of used AGD bathwater (2 h, 4 h, 6 h or 8 h) or unused freshwater (negative control) was added to triplicate tanks to achieve a final inoculation salinity of 30 ppt (Figure 1 and Figure S1) and a stocking density of approximately 49 kg/m^3^. The fish were then monitored in the 300 L static volume with constant aeration and supplementary oxygen. After two hours, the seawater inlet on each tank was returned at 1 L/min (0.12 tank volumes/h), allowing the tank to slowly fill over the next two hours, at which stage the seawater flow was returned to 7 L/min (0.8 tank volumes/h) to overflow through the outlet and gradually displace the remaining bathwater. This exposure period was intended to allow at least 4 h for surviving pseudocysts to return to the trophozoite stage. 

The tank lids remained closed and all equipment (dipnets, buckets, water quality sampling, etc.) was kept separate to minimize the risk of contamination between treatments during holding of naïve fish, inoculation and the subsequent monitoring phase out to 40 days post inoculation (dpi).

### 2.3. AGD Development in Fish Exposed to Used AGD Bathwater

Following the used AGD bathwater inoculation, the fish (29.4 kg/m^3^) were held in 35 ppt seawater at 15.4 ± 0.16 °C with constant aeration (93.2 ± 6% oxygen saturation) and fed to satiation (intake 0.95 ± 0.18% body weight/day), with daily observation for signs of developing AGD (listless fish, opercular flaring, morbidity with gross gill signs).

At 20 dpi, 5 fish per tank were haphazardly dip netted and transferred to 100 mg/L Aqui-S for terminally sampling. This was performed in reverse order of AGD risk (starting with negative control, then 8, 6, 4 and 2 h) and sampling equipment was kept separate in order to minimize any risk of contamination between treatments. Adjacent tank lids remained closed while fish were being sampled, and these barriers were supplemented by tarpaulin covers. Anesthetic bins and work surfaces were cleaned and disinfected (2% Virkon^TM^ in freshwater, freshwater rinsed and drained) between each sampled tank. Each sampled fish was weighed and gill scored [21,37]. Then, the four right-side gill arches were swabbed using sterile disposable cotton swabs (Westlab) and preserved in RNAlater (Life Technologies). Finally, the first and second gill arches from the left and right-hand sides (L1, L2, R1, R2) were excised and fixed in Davidson’s seawater fixative at a fixative:tissue ratio of 10:1 for 24 h. 

At this stage, it was evident that all sampled fish in the 2 h bathwater exposed group displayed advanced AGD pathology. It was considered unlikely that the remaining fish in this treatment could continue to 40 dpi without significant adverse fish welfare impact, and therefore the decision was taken to terminate the remaining fish in these 3 tanks (total 30 fish). An additional 5 fish per tank were sampled as described above and the other 5 fish per tank were gill scored and weighed.

The remaining treatments (4, 6 h, 8 h and negative control) comprising 10 fish per tank (30 per treatment) were held for a further 20 days until trial termination at 40 dpi. As previously described, 5 fish per tank were sampled for weight, gill score, gill swab and gill histology. The remaining 5 fish per tank were weighed and gill scored.

### 2.4. Gill Histology

After 24 h in Davidsons fixative, the four preserved gill arches per individual were photographed (Canon 300D, Canon, Tokyo, Japan) in a light box, then the L2 holobranch was transferred to 70% ethanol and stored until processing. L2 underwent dehydration and wax infiltration using an automated tissue processor (Leica TP1020, Wetzlar, Germany). Following this, samples were embedded in paraffin wax and the anterior hemibranch surface was sectioned at 5 µm using a Leica RM2235 microtome (Leica, Wetzlar, Germany). Sections were de-waxed at 60 °C, stained with haemotoxylin and eosin using standard histology protocols and cover-slipped. Samples were investigated using a light microscope (Leica DM1000, Wetzlar, Germany), and observed for characteristic AGD lesions with associated *N. perurans* [19]. Areas of non-normal gill morphology were assessed, and the number of filaments containing areas of lesion or plaques recorded to give a proportion of affected filaments across each hemibranch surface. All slide photography was completed using a digital microscope camera (Luminoptic ISH500, Tucsen Photonics, Fuzhou, China).

### 2.5. Quantification of N. perurans in Water Samples—Viability Assay

Water samples were taken from the CIT seawater prior to removal of AGD-affected fish for bathing, from the used AGD bathwater at 2, 4, 6 and 8 h and from the control (unused) freshwater. For each treatment group, 3 × 50 mL sterile Falcon^®^ tubes were submerged approximately 0.1 m sub-surface, passed through a 1000 μm stainless steel screen to pre-filter large debris. The 3 tubes represented 3 biological replicates. Samples were gently spun down (2500 rpm) for 10 min and the supernatant was poured off from each tube to leave ~1 mL of suspension. The suspensions were then vortexed briefly to homogenize, before 1 mL of each suspension was aliquoted into a 1.5 mL tube. These tubes were stored at −80 °C until DNA extraction and *N. perurans* qPCR assays were completed.

DNA was extracted from 800 μL of the 1 mL cell suspension using a DNeasy Blood and Tissue kit (Qiagen, Hilden, Germany). The 800 μL cell suspension was first incubated with 180 μL of ATL buffer (Qiagen) and 20 μL of Proteinase K (20 mg/mL) (Qiagen) for 4 h at 56 °C. Following this incubation, the DNA was extracted exactly as per the DNeasy Blood and Tissue kit manufacturer’s protocol. DNA was eluted in 50 μL of Qiagen elution buffer. Quantitative PCR was performed targeting the 18S rRNA gene of *N. perurans* using the methods previously described [38] in a 10 μL reaction volume. Triplicate PCR reactions were performed for each biological replicate water sample and the mean Ct (cycle threshold) was calculated. 

### 2.6. Molecular Analysis of Gill Swabs

Gill swabs in RNAlater were stored at 4 °C for 24 h and then at −20 °C until DNA extraction. The swabs were shaken in a FastPrep-24™ 5G lysis system (MPBio, Irvine, CA, USA) at 6 m/s for 40 s. After a pulse centrifuge, the swab was removed and transferred to a clean tube using sterile forceps, taking care not to cross-contaminate samples. Samples were then spun down at 14,000 rpm for 10 min in order to form a visible pellet and to facilitate removal of RNAlater. DNA was extracted from pellets and swabs using the Wizard^®^ SV 96 Genomic DNA Purification System (Promega, Madison, WI, USA) as per the manufacturer’s protocol and combined. Real-time quantitative PCR was performed on purified DNA (normalized to 10 ng DNA per 10 μL qPCR reaction), targeting the 18S rRNA gene of *N. perurans* using the methods previously described by [38,39]. The Ct values were normalized to the endogenous control gene salmon elongation factor (ELF) as described previously and a threshold limit of 40.13 was applied [38].

### 2.7. Statistical Analysis

In order to test for the effect of treatment dose on gill score development, we first analysed the effect of ‘tank’ and ‘treatment’ factors using a non-parametric Scheirer–Ray–Hare extension of the Kruskal–Wallis test for all gill scored fish (5 per tank, 15 per treatment from the 4, 6, 8 h and negative control treatments at Day 20; 15 per tank from the 2 h treatment on Day 20; 10 per tank from the 4, 6, 8 h and negative control treatments at Day 40). ‘Tank’ was not found to be a significant factor, and therefore for downstream analysis, tank replicates were combined within treatment for both sampling days. A Wilcoxon pairwise test with a Benjamini–Hochberg correction was used to compare between ranked treatment groups at each timepoint. 

Histology data identified as non-normal using a Shapiro–Wilk test underwent arcsine transformation prior to statistical analyses. A two-way ANOVA was used to compare the proportions of affected filaments between treatment groups, and timepoint (20 and 40 dpi). Tukey’s HSD test was used to assign significant interaction effects post hoc. All figures were produced in R version 3.6.1 [40] using the ggplot package [41].

## 3. Results

### 3.1. Confirmation of AGD in CIT Fish

**Figure 2:**
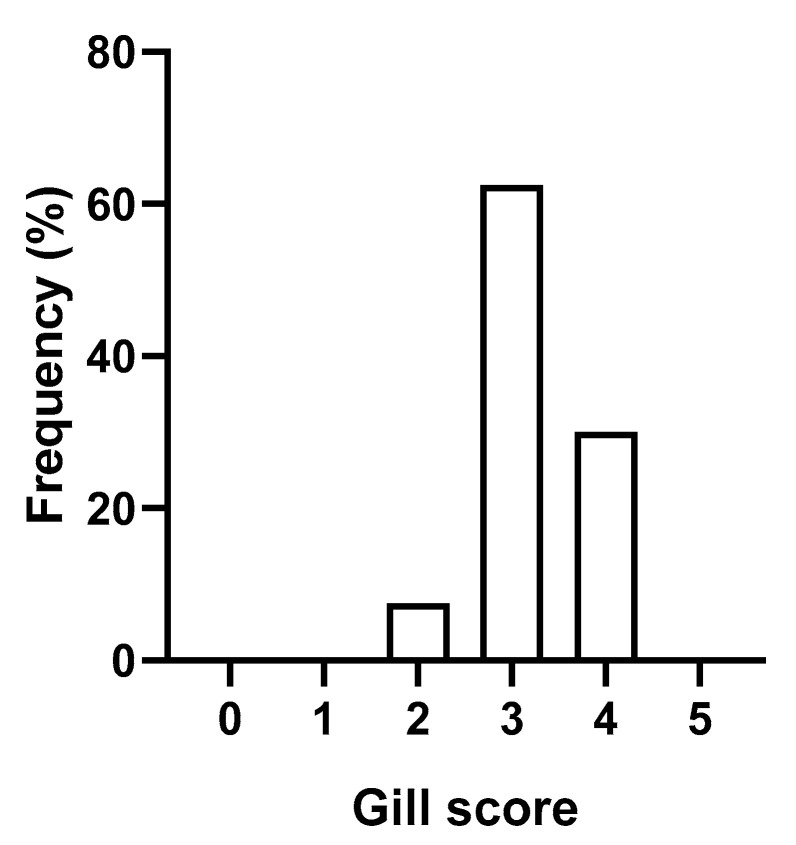
Gross gill score distribution of CIT fish (n = 40), assessed immediately after removal from a 2 h freshwater bath treatment.

Advanced AGD was evident in the CIT fish that were used to produce gill-detached *N. perurans* by freshwater bathing. An average gill score of 3.22 ± 0.09 (mean ± SEM) was obtained immediately post treatment (Figure 2). The presence of *N. perurans* was confirmed by gill swab qPCRs in all five of the CIT fish that were sampled (mean Ct = 37.37 ± 0.62).

### 3.2. N. perurans Loads in Pre-Inoculation Water Samples

Water samples (50 mL) taken from CIT seawater immediately prior to removal of fish for freshwater treatment were positive for DNA of *N. perurans* (mean Ct = 30.56 ± 0.41). 

**Figure 3:**
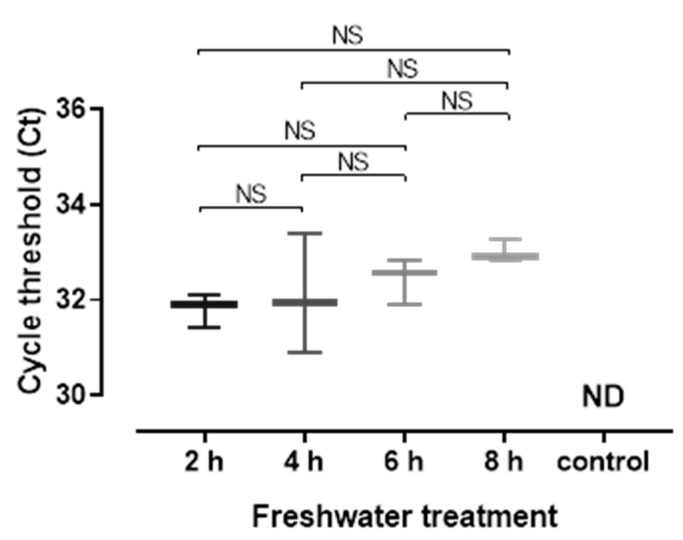
Quantitative RT-PCR results from 50 mL samples of AGD bathwater taken immediately following treatment of AGD-affected CIT fish (“2 h”) or following holding for a further 2 h (“4 h” treatment), 4 h (“6 h” treatment) or 6 h (“8 h” treatment). *N. perurans* DNA was not detected in the unused freshwater control (ND). Box and whiskers plot shows median value (horizontal bar); vertical bars show range of values (samples n = 3 per treatment). NS: not significant.

DNA of *N. perurans* was detected at each timepoint in used AGD bathwater (Figure 3). However, there was no significant difference in *N. perurans* load (based on Ct) between treatment timepoints (One-way ANOVA, F (3,8) = 1.60, *p* = 0.2634). *N. perurans* DNA was not detected in the negative control freshwater samples.

### 3.3. Gill Score Progression in Bathwater Exposed Fish

**Figure 4:**
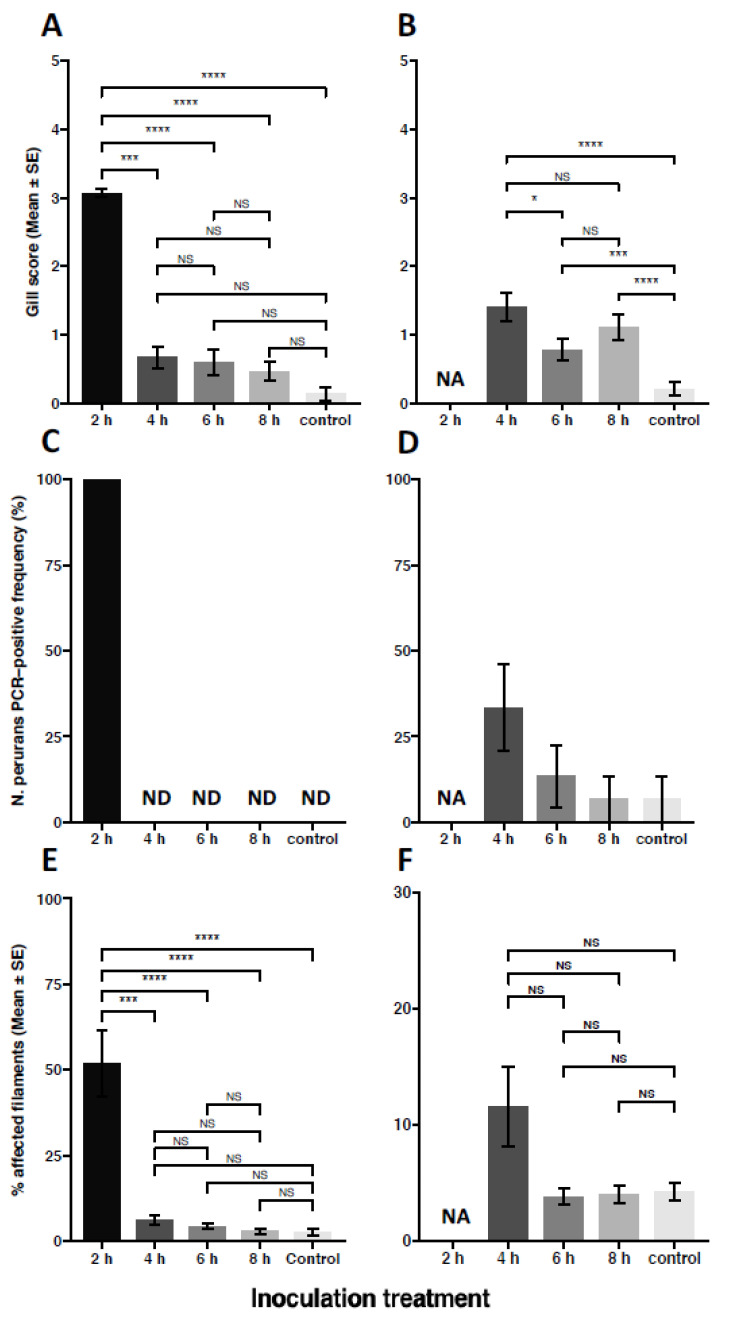
Comparison of AGD signs at 20 and 40 days post inoculation (dpi) with used AGD bathwater or freshwater at 15 °C and 30 ppt. (**A**) Mean gross gill score at 20 dpi (n = 5 fish per tank, 15 per treatment for the 4, 6, 8 h and negative control treatments; 15 fish per tank, 45 total for the 2 h treatment. Gill score, determined according to Taylor et al. [21], was significantly higher in the 2 h treatment than all other treatments. By 40 dpi (**B**), gill scores in the remaining groups remained low but significant differences were seen between AGD bathwater exposed treatments (4, 6 and 8 h) and the control group (n = 5 fish per tank, 15 per treatment). The frequency (%) of positive PCR detection of *N. perurans* per treatment group is shown in (**C**) at 20 dpi (n = 5 fish per tank for the 4, 6, 8 h and control treatments, 10 fish per tank for 2 h treatment). Molecular detection of *N. perurans* was confirmed in all 2 h fish at 20 dpi but was not seen in the other treatment groups (ND = not detected). By 40 dpi (**D**) *N. perurans* was detected at lower frequency in all treatments (n = 5 fish per tank), including a single negative control individual. A comparison of mean lesion affected filaments determined by histopathology is shown in (**E**) at 20 dpi and (**F**) at 40 dpi (n = 3 per tank, 9 per treatment at both timepoints). AGD was definitively confirmed in the 2 and 4 h treatments at 20 dpi, and the 4 h treatment at 40 dpi via presence of *N. perurans* trophozoites adjacent to areas of lamellar fusion and hyperplasia. The 2 h bathwater produced higher proportions of AGD-affected lamellae at 20 dpi and was significantly different from all other treatment groups at both 20 and 40 dpi). No other significant interactions were observed. NA = not available. All fish in the 2 h treatment were ethically killed at 20 dpi due to high average gill score, and therefore the 2 h treatment was not represented at 40 dpi. Lines connect two categories where the differences were significant with * (*p* < 0.05), *** (*p* < 0.001) or **** (*p* < 0.0001). Bars show the mean ± standard error of the mean (SEM).

Although no AGD related mortalities occurred throughout the trial, typical AGD gross gill signs (raised white spots and patches) were assessed at a mean gill score of 3.07 ± 0.06 by 20 dpi (Figure 4A) in fish that had been inoculated with 2 h used AGD bathwater. While there was no statistically significant interaction between tank and treatment (SRH KW, df(210), H = 83.980, *p* = 1.006), the effect of treatment alone appeared to most significantly affect gill score (df(4), H = 124.056, *p* < 0.00001) while tank alone did not affect gill score (df(10), H = 15.964, *p* = 0.10066). A subsequent comparison of timepoints indicated that gill score was significantly higher in the 2 h exposed fish at 20 dpi (*p* < 0.0001) compared to the other treatment groups, while there were no significant differences between 4, 6, 8 h and negative control gill scores (Figure 4A). The gill score of the 2 h bathwater inoculum-exposed fish at 20 dpi was also significantly greater than the gill score in all other treatments at 40 dpi (*p* < 0.0001). The 2 h treatment was terminated at 20 dpi due to high gill score, as specified in our animal welfare procedures.

A significant effect of treatment was evident at 40 dpi across the remaining treatment groups (df(3), X^2^ = 25.287, *p* < 0.0001), with a significant effect of tank (df(11), X^2^ = 44.246, *p* < 0.0001). AGD signs were significantly higher in the remaining groups that had been exposed to bathwater inoculum (4, 6 and 8 h treatments, Figure 4B) when compared to the control (unused freshwater) exposed fish (Wilcox; *p* < 0.0001, *p* < 0.001, *p* < 0.0001, respectively). The 4 h bathwater inoculum-exposed treatment had a higher gill score than the 6 h treatment (*p* = 0.03613) but were not significantly different to the 8 h treatment (*p* = 0.20149). Importantly, the 4 h treatment at 40 dpi was significantly higher than the same treatment at the previous 20 dpi timepoint (*p* < 0.05).

### 3.4. Molecular Analysis of Gill Swabs

All of the 2 h bathwater inoculum-exposed fish sampled at 20 dpi were positive for *N. perurans* DNA (Figure 4C), with Cts ranging between 36.8 and 29.9 (mean 33.3, data not shown). There were no positive qPCR detections of *N. perurans* in samples from the 4 h, 6 h, 8 h or negative control treatments at 20 dpi.

At 40 dpi (Figure 4D), positive qPCR detection of *N. perurans* occurred in 33.3% of the fish sampled from the 4 h treatment, with Cts ranging between 37.8 and 29.9 (mean 34.8). However, these detections were only made in one of the three replicated tanks (A2, all 5 sampled fish) indicating a lack of consistency of infection dynamics between tanks. From the 6 h treatment, 20.0% of samples (3 fish) were positive for *N. perurans* with detections occurring in two of the three replicate tanks. Cts in the 6 h group ranged between 38.21 and 36.70 (mean 37.65). A single positive detection was made in the 8 h group (6.7%, Ct 39.75). 

A single positive qPCR detection (6.7%, Ct 37.7) was made from the negative control treatment. Although we cannot discount this result, a review of our records confirms that this is most likely due to laboratory contamination. At the DNA extraction phase, samples were loaded in chronological (sampling) order onto a 96 well plate. This placed the thirty positive 2 h (20 dpi) samples across two lanes (lane F and G) and included the first six positions in lane H. The last six positions (H7 to H12) were taken up by the first six negative control samples from the 40 dpi sampling. Our questionable sample was adjacent to three strong positives (Ct values 31.7–29.9). Although extraction controls were not included on the plate, it is feasible that aerosol contamination may have occurred during pipetting of samples. The remaining 54 samples from 40 dpi and the five post bath CIT samples were loaded to a separate extraction plate. For the PCR analysis, triplicate samples were randomized across ten 96 well plates, each plate contained duplicate non-template controls (NTC) for *N. perurans* and two for ELF. All NTC’s failed to amplify, indicating no reagent contamination.

### 3.5. Gill Histopathology

**Figure 5:**
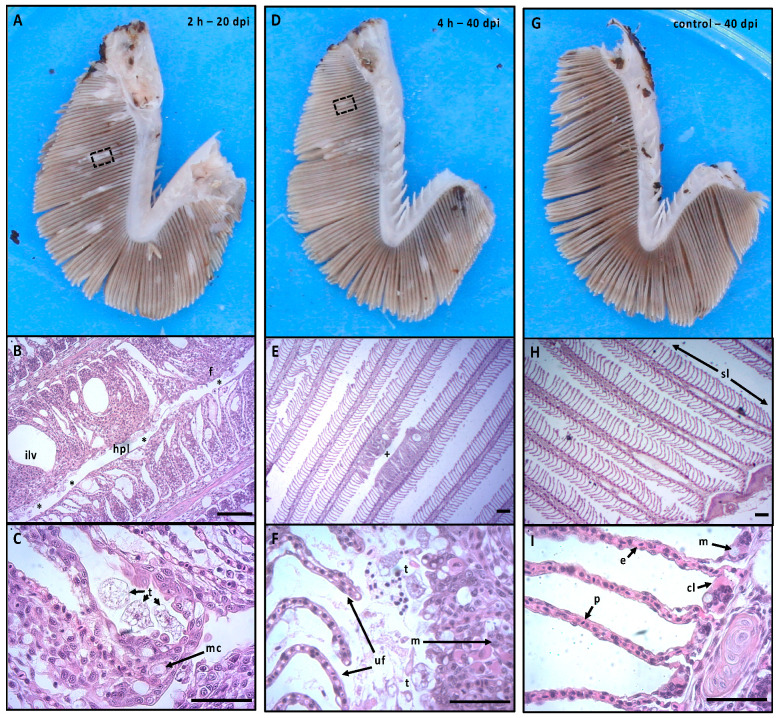
Representative gross imagery and histology from AGD-affected (2 h at 20 dpi, 4 h at 40 dpi) and AGD-unaffected (control 40 dpi) treatments. (**A**) Gross image of Davidson’s fixed gill hemibranch from heavily AGD-affected fish from 2 h treatment group. Black inset box shows multifocal lesioned area. (**B**) shows higher magnification of highlighted area in (**A**), depicting pronounced hyperplasic lesion (hpl) characterized by fusion (f) of the secondary lamellae, formation of interlamellar vesicles (ilv), and high abundance of *N. perurans* trophozoites (*), scale bar = 100 μm. (**C**) 400× magnification of lesion margin of 2 h fish, with *N. perurans* trophozoites accumulated between interlamellar spaces (t), and high numbers of mucus cells (mc) along the proliferative margins, scale bar = 50 μm. (**D**) Gross image of lightly affected hemibranch surface from the 4 h treatment group. (**E**) Higher magnification (25×) of small lesion (+) of approximately 10 interlamellar units highlighted (dashed inset box) in (**D**), scale bar = 200 μm. (**F**) 400× magnification of lesion showing area of lamellar fusion infiltrated with white bloods cells including macrophages (m) and associated *N. perurans* trophozoites (t) adjacent to unaffected adjacent lamellae (uf), scale bar = 50 μm. (**G**) Gross image control unaffected fish hemibranch clear of typical AGD signs. (**H**) Unaffected gill at 25× magnification depicting anatomically normal secondary lamellae (sl), scale bar = 200 μm. (**I**) 400× magnification of anatomically normal secondary lamellae showing pillar cells (p), chloride cells (cl), erythrocytes (e) and mucus cells (m), scale bar = 50 μm.

A significant treatment (two-way ANOVA, F (3,8) = 4.075, *p* < 0.05) and tank (*p* < 0.01) effect was observed at 20 dpi, though the tank effect was confined to the 4 h bathwater inoculation treatment. AGD was definitively confirmed in fish exposed to the 2 and 4 h bathwater inocula at the 20 dpi sampling, with areas of hyperplasic lesions and fusion of secondary lamellae with adjacent *N. perurans* trophozoites associated with the proliferative margins (Figure 5C,F). AGD lesions were characterized by hyperplasia of the lamellar epithelium and infiltration with white blood cells including leukocytes. Fish from the 2 h treatment showed advanced signs of AGD (mean affected filaments 51.72% ± 9.62, range 20–96%), with 3 individuals from the 9 examined arch samples exhibiting > 80% of affected filaments. The 2 h treatment group at 20 dpi was significantly different to all other groups at both timepoints (Figure 4E, F), although the 2 h fish were proactively terminated prior to reaching 40 dpi. Histologically, the 4 h treatment group showed signs of light AGD at 20 dpi, with a small number of lesions identified (6.14% ± 1.40). Although the percentage of lesioned filaments from the 4 h samples (across all 3 replicates) were not significantly different to the 6, 8 h and control samples, a significant (*p* < 0.01) tank effect was evident within the 4 h treatment whereby tank A2 presented with 10.68% ± 1.19 lesioned filaments. Importantly, despite the negative qPCR results reported for the 4 h 20 dpi fish above, occasional amoeba trophozoites were observed within the 3 fish sampled from this tank. 

Despite the presence of a low level of non-normal gill pathology in the 6 and 8 h samples at 20 dpi (4.26% ± 0.88 and 2.76% ± 0.86 affected filaments, respectively) AGD could not be positively confirmed as amoebae trophozoites were not seen on these slides. The lesions observed were typically characterized by small nodules (1–5 interlamellar units) demonstrating lymphocytic infiltration (Figure S2). 

No typical AGD gill pathology was observed in negative control fish, although there was a small amount of interlamellar fusion in parts of the gill affected filaments (2.62% ± 0.96). Importantly, none of these areas of non-normal pathology were observed with associated amoebae trophozoites.

At 40 dpi (Figure 4F), a significant treatment (two-way ANOVA, F (3,8) = 4.611, *p* < 0.05) and tank (*p* < 0.01) effect was also observed. The overall percentage of lesioned filaments in the 4 h bathwater inoculum treatment was 11.55% ± 3.43, but this was not significantly different to the 4 h 20 dpi measure or the 6 h, 8 h or control treatments at 40 dpi. This is due to inter tank variability within the 4 h group, with tank A2 presenting higher proportions of affected filaments (24.35%) comparative to tank replicates A13 (5.08%) and A15 (5.21%). As observed at the 20 dpi measure, *N. perurans* trophozoites were only seen within tank A2. Similarly, trophozoites were not seen in association with lesions in the 6 h or 8 h treatments despite 20% and 6.7% positive detection of *N. perurans* DNA by qPCR. Despite a single positive qPCR detection of *N. perurans* DNA in the negative control treatment, no trophozoites were observed by histopathology on this individual or across the 9 samples examined from this treatment.

## 4. Discussion

The primary aim of parasitic disease treatment in intensive fish culture is to reduce or remove the parasite load, thus promoting improved health, production, and animal welfare. Modern integrated pest management approaches include initiatives to prevent the release of viable parasites back into the aquatic environment following antiparasitic treatment. These approaches are essential to minimize the infective load in the marine environment and underpin strategies to rapidly treat an entire farm without infecting already treated or naive populations nearby. Removal of detached sea lice by filtration of treatment discharge water is now increasingly common on well boats [14] after physical (thermal/water jet), chemical or freshwater delousing, but similar strategies have yet to be considered to prevent the release of detached amoebae following AGD freshwater treatment. In this study, we have demonstrated that gill-detached trophozoites were able to survive a commercially relevant 2 h freshwater bath of AGD-affected fish at 72 kg/m^3^ and recover in 30 ppt seawater to cause extensive AGD lesions (gill score 3.1, 51.7% affected filaments) in naïve salmon. These gill lesions were histologically typical of AGD, with high numbers of associated trophozoites (Figure 5B) and DNA of *N. perurans* was detected in all gill swab samples. The rapid development of extensive macroscopic gill lesions reported here is consistent with the results of Crosbie et al. [42], who challenged naïve fish held at 16–16.5 °C with 500 cells/L *N. perurans* isolated from the gills of AGD-affected (CIT) fish and reported development of extensive gill lesions and the onset of AGD related mortality at around 23 days post challenge. Adams et al. [43] reported ~25% AGD lesion affected filaments by 32 days (15.4 °C) following 100 cells/L challenge. Slower AGD development occurs with lower viable *N. perurans* infective dose, as shown by Bridle et al. [44], who recorded 58–60% affected filaments at 64 days following infection by 10 *N. perurans*/L at 15–16.5 °C. Our study clearly demonstrates that a significant number of amoebae can survive 2 h freshwater treatment and are infective when returned to seawater. This result suggests that commercial AGD bath treatment for 2 h following the transfer of the last fish into a freshwater tarpaulin or wellboat risks the return of viable amoebae into the marine environment. 

As a free-living parasite, *N. perurans* infection of farmed Atlantic salmon initially occurs from the marine environment, but once established on a farm the production and shedding of trophozoites from infected fish rapidly amplifies the trophozoite load in the water column. A higher prevalence of *N. perurans* in the vicinity of AGD-affected salmon pens has previously been demonstrated, with concentrations ranging between 0–62 cells/L [27,28] and it is likely that the number of amoebae shed is proportional to the infection intensity [45] and affected fish biomass. There is also evidence that current 2–3 h freshwater treatments do not clear all amoebae from the gills [24,25,26], with surviving trophozoites acting as a source of reinfection within treated populations. Our study establishes the possibility of a further source of infection (or reinfection) through the release of surviving amoebae which can return to infective trophozoites within hours of their return to seawater. The relative contribution of each of these infection sources to AGD development within farmed salmon populations likely varies with a range of biotic and abiotic factors.

In order to develop a simple strategy to limit the return of viable amoebae after freshwater treatment, our study examined whether holding used bathwater for an extended period would lower the infective dose of viable amoebae being released into seawater. Previous in vitro studies have shown that freshwater exposed *N. perurans* rapidly detach as floating pseudocysts, which recover to trophozoite form when returned to seawater and subsequently attach to plastic substrates. According to Wright et al. [34] losses are negligible following short-term exposure to freshwater (2 to 30 min) when assessed 1 h after return to seawater, but no amoebae recovered after 1 h at 35 ppt following a 1 or 2 h freshwater exposure. However, it is probable that pseudocysts surviving an extended freshwater treatment will take a longer time to recover when returned to seawater. Lima et al. [33] collected remaining floating cells after 2 h of freshwater exposure and observed that reattachment began between 3 and 5 h following return to 35 ppt at 15 °C, with 16% of those cells surviving after 24 h. Amoebae exposed to freshwater for shorter periods (30 min and 1 h) began to recover more rapidly, with 87% and 77% surviving after 24 h, while only 4.3% of the floating cells survived after 3 h freshwater exposure and none survived following 4 h exposure. In our in vivo study, exposure to 4 h bathwater inoculum caused AGD to develop in 1 out of 3 tanks. At 20 dpi, AGD in the 4 h exposure was demonstrated by the development of small histologically characteristic gill lesions with infrequent associated amoebae (Figure 5F), although gross gill score was not significantly different to the 6 h, 8 h or control treatments. Surprisingly, *N. perurans* DNA was not detected in these fish despite visual confirmation of their presence. This may indicate that the distribution of *N. perurans* was inconsistent between the swabbed gill arches (R1 to R4) and the histological sections from L2, and/or the overall load of amoebae on the gills was low. By 40 dpi, gill score had increased in the 4 h treatment (Figure 4B), *N. perurans* DNA was detected and amoebae were readily seen in association with lesioned gill filaments, confirming the ongoing development of AGD. However, AGD was only confirmed in one tank (A2), and this lack of consistency between 4 h replicates suggests a sparse and inconsistent availability of viable amoebae. The variation between the replicate tanks is not unexpected given that these fish were likely exposed to viable amoeba loads that were approaching the minimal infectious dose.

The results following longer holding of used bathwater suggest low and patchy infectivity by surviving amoebae. Although gill score increased slightly between 20 and 40 dpi in the 8 h treatment, there were no significant changes in the 6 h treatment. Histopathology detected a low level of non-normal gill pathology in both treatments at 20 and 40 dpi, but these lesions were not confirmed as AGD because amoeba trophozoites were absent. However, positive detection of *N. perurans* in 20% of the 6 h samples at 40 dpi confirmed that amoebae were present. A single fish tested positive for *N. perurans* in the 8 h treatment, though at a high and marginal Ct (39.75, compared to threshold of 40.13). Interpretation of these results is complicated by the finding of a single *N. perurans* DNA positive individual from a negative control tank at 40 dpi. This individual was assessed at gill score 0 and the gill did not exhibit any AGD signs by histopathology. Having reviewed laboratory procedures, we believe that this finding possibly occurred through contamination from 2 h 20 dpi samples during DNA extraction as the samples were processed together. We acknowledge that this possible contamination lowers confidence in the 40 dpi results in the other treatments. Notwithstanding the uncertainty raised, the results presented here demonstrate that amoebae surviving bathwater exposure are capable of causing AGD. The precise duration at which the amoebae lose their infectious potential following prolonged exposure to freshwater beyond 2–4 h needs to be further evaluated. Nevertheless, it appears that increased holding time before disposal of used AGD bathwater will lower the infectivity of remaining amoebae and that the risk becomes negligible after 4 h. This occurred despite the fact that a consistent *N. perurans* DNA load was observed in the used bathwater between 2 and 8 h. Taken together, this finding suggests that while the load of amoeba remained constant in the bath water, the viability and thus infectious potential of the amoeba decreased over the time course. This finding also highlights the limitations of the traditional 18S PCR to discriminate between viable and non-viable amoebae.

Due to experimental logistics and animal welfare constraints, it may be argued that our 30 ppt static inoculation protocol was not fully representative of commercial conditions where AGD bathwater is released directly into full strength seawater. Freshwater is less dense, so it tends to disperse across the surface for some time before being diluted and mixing with seawater. This process arguably presents a more rapid salinity transition (from 0 ppt to 35 ppt) which may be more stressful to pseudocysts than our 30 ppt experimental protocol. It is also probable that our inoculation procedure (2 h at 60% static volume, followed by 2 h of dilution, then flushing out between 4 and 6 h) may not have allowed enough time for the gradual morphosis between pseudocyst and settled trophozoite forms, which is likely more prolonged after extended freshwater exposure [33]. However, as significant water dispersion will occur over 4 or more hours after freshwater is released in the marine environment, it is unlikely that a longer incubation time would be relevant to assess the localized risk of bathwater disposal. Further work to assess viability after more prolonged pseudocyst recovery in seawater would support modelling of viable parasite dispersion over longer time periods. Previous studies have shown that farm sites may be highly connected following release of small pathogen particles over longer dispersion times [7,46,47]. 

Our water source for this study was moderately hard (125 mg/L GH) water. Previous work [32] has shown that amoebae exposed in vitro to water containing high concentrations of Ca^2+^ or Mg^2+^ (200 mg/L) showed high levels of survival up to 3 h of exposure. Experiments conducted in the laboratory and on-farm [48] have shown that soft fresh water (19.3–37.4 mg/L CaCO_3_) is more efficacious for bathing marine AGD-affected Atlantic salmon than hard fresh water (173–236.3 mg/L). This improved bathing method significantly reduced viable gill amoebae remaining within gills and proved to be of a therapeutic benefit by alleviating the pathological signs of AGD. Therefore, it is likely that survival of viable amoebae in used bathwater could be reduced by softer freshwater sources.

Although prolonged holding of used bathwater is a logical approach, it may not be logistically effective on commercial farms where multiple pen populations need to be treated each day. Therefore, more rapid ways to remove viable amoebae before releasing used bathwater could be explored. As pseudocysts are turgid with freshwater, they will likely be fragile and easily disrupted by physical or chemical intervention once fish have been removed from the water. Exploration of these methods can be supported now that we have a successful in vivo bathwater inoculation challenge method. As in vitro studies have already indicated that some amoebae can survive hydrogen peroxide AGD treatment and are able to uptake neutral red dye when returned to normal seawater [49], this experimental inoculation method could be utilized to explore whether surviving *N. perurans* retain their infectivity following alternative AGD treatments.

Not only does the release of viable amoebae into the marine environment pose a localized risk of infection, there is concern that freshwater resistant *N. perurans* strains may develop over time because freshwater resistant strains may outcompete susceptible amoebae [34]. Evidence of increased frequency of freshwater bathing over the first 20 years of the Tasmanian salmon industry may suggest that freshwater exposure was becoming less effective [26,34]. However, this is likely also related to increased salmon biomass and farm proximity along with a shift to more proactive bath treatments at lower average gill score. Indeed, with the development of the industry selective breeding program which has targeted increased AGD resistance there has been a decrease in bathing frequency over the past decade [50]. The likelihood that a proportion of amoebae have survived commercial AGD treatment over the past 35 years raises a logical concern that *N. perurans* can adapt to a more freshwater resistant status, but there is little empirical evidence to support or refute it. Further research is required to characterize whether differential freshwater tolerance exists between *N. perurans* strains and to characterize genetic differences that may be linked to this tolerance.

## 5. Conclusions

Our study has established that a standard commercial freshwater treatment (in which AGD-affected salmon are held in freshwater for 2 h after the last fish is transferred to the treatment enclosure) is likely to release a concentration of infective amoebae back into the marine environment. The survival of *N. perurans* following freshwater exposure raises concern that amoebae may adapt resistance to AGD bath treatment, though further work is required to explore this.

With the aim of supporting development of integrative pest management techniques to improve AGD management, we explored whether holding used bathwater for an extended period would reduce its infectivity. This is a technique that could be applied on a wellboat (after pumping out and dewatering treated fish) to retain the used bathwater in the ship or by transferring to a tarpaulin liner or pond for an effective period. Following an initial 2 h treatment, we found that water retained for an additional 2 h (i.e., 4 h bathwater) produced low numbers of *N. perurans* on gills and early AGD pathology visible by histopathology at 20 dpi, yet there was no detection of *N. perurans* DNA. By 40 dpi, AGD was established and *N. perurans* were readily detected. This response was variable between fish and tanks, as described by Bridle et al. [44] following experimental inoculation with low numbers of infective amoebae (1 cell/L). 

Longer holding times (6 and 8 h bathwater) produced patchy results (low level *N. perurans* DNA detection and inconclusive pathology) suggesting that the viability and infectious potential of amoeba was decreased with longer holding time. The variability of results for retained water reported here indicate that the viable load was reduced to at or below a minimum infective dose. However, due to the positive detection of *N. perurans* DNA in a single negative control fish at 40 dpi, further work is warranted to fully validate these results.

## Supplementary Materials

The following are available online at https://www.mdpi.com/article/10.3390/microorganisms9050967/s1, Figure S1: Water quality parameters during the AGD used bathwater and control inoculation. (A) Salinity ppt (B) Oxygen % saturation. Figure S2: Gross images representative of 6 h (A) and 8 h (D) bathwater challenged fish (at 40 dpi) with no apparent AGD expression.
