# Supplementary material for: Evaluation of the Infectious Potential of Neoparamoeba perurans Following Freshwater Bathing Treatments
Source: Microorganisms. 2021 Apr 29;9(5):967. doi: 10.3390/microorganisms9050967 (PMC8145359; doi:10.3390/microorganisms9050967)
Supplement: Supplementary file 1 [file microorganisms-09-00967-s001.zip › microorganisms-1185869-SI.pdf]

microorganisms

Article

Evaluation of the Infectious Potential of *Neoparamoeba perurans* Following Freshwater Bathing Treatments

Richard S. Taylor [1]*, Joel Slinger [2,3], Chris Stratford [2], Megan Rigby [1] and James W. Wynne [1]

[1] CSIRO Agriculture and Food, Castray Esplanade, Hobart, TAS 7000, Australia; megan.rigby@csiro.au (M.R.); james.wynne@csiro.au (J.W.W)
[2] CSIRO Agriculture and Food, Bribie Island Research Centre, Woorim, QLD 4507, Australia; joel.slinger@csiro.au (J.S); chris.stratford@csiro.au (C.S)
[3] Institute for Marine and Antarctic Studies, University of Tasmania, Launceston, TAS 7250, Australia; joel.slinger@utas.edu.au
* Correspondence: richard.taylor@csiro.au; Tel: +61-3-62-325-089 (R.S.T)

Citation: Taylor, R.S..; Slinger, J.; Stratford, C.; Rigby, M.; Wynne, J.W. Evaluation of the Infectious Potential of Neoparamoeba perurans Following Freshwater Bathing Treatments. *Microorganisms* **2021**, *9*, 967. https://doi.org/10.3390/microorganisms9050967

Academic Editor: María Teresa Gómez-Muñoz

Received: 31 March 2021
Accepted: 27 April 2021
Published: 29 April 2021

Publisher's Note: MDPI stays neutral with regard to jurisdictional claims in published maps and institutional affiliations.

Copyright: © 2021 by the author. Licensee MDPI, Basel, Switzerland. This article is an open access article distributed under the terms and conditions of the Creative Commons Attribution (CC BY) license (http://creativecommons.org/licenses/by/4.0/).

Supplementary materials

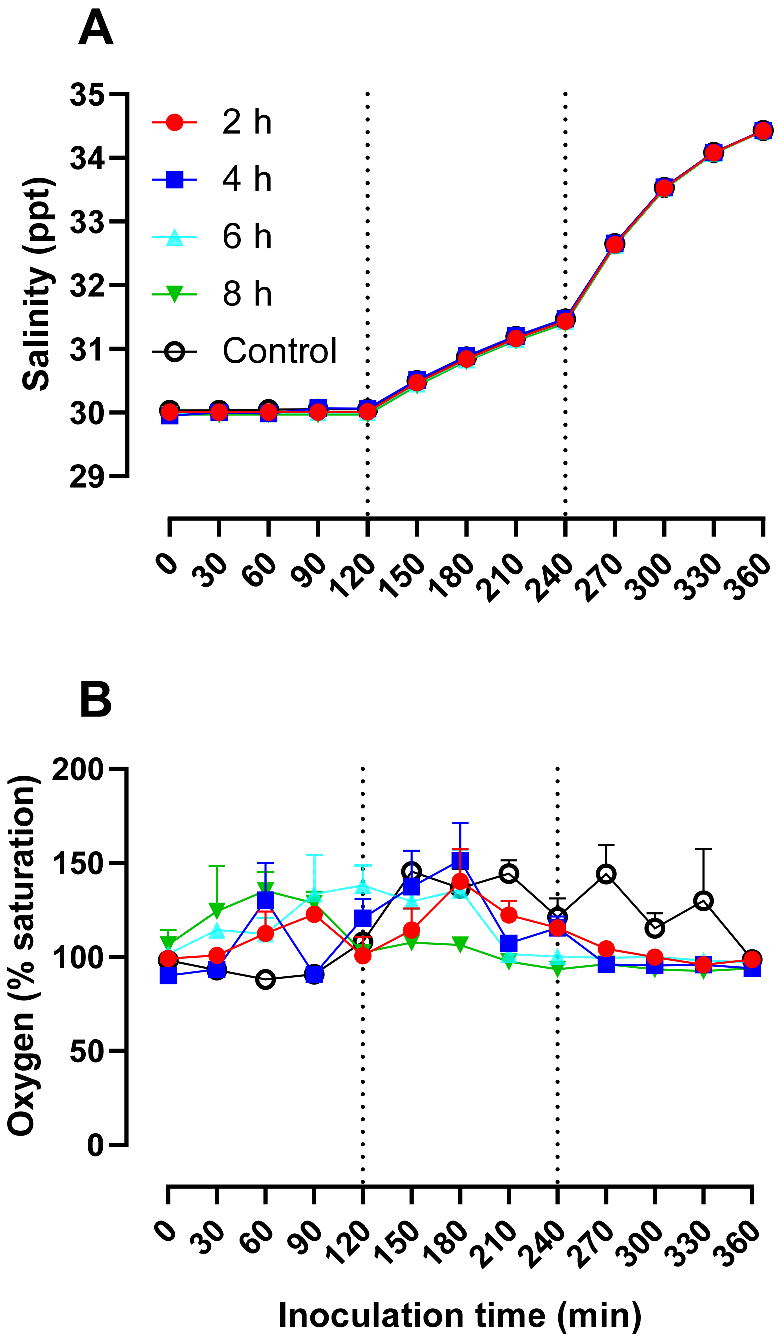

Figure S1. Water quality parameters during the AGD used bathwater and control inoculation. (**A**) Salinity of 30 ppt was created by mixture of 40 L of bathwater/freshwater in 260 L of 35 ppt seawater. After 120 min seawater was introduced at 1 L/min, allowing the tank to slowly fill and salinity gradually increased. From 240 min, seawater flow was increased to 7 L/min. Error bars are not visible due to low variation between tanks. (**B**) Oxygen was controlled by constant aeration and supplementary oxygenation (manual control). Mean + standard error of the mean (SEM).

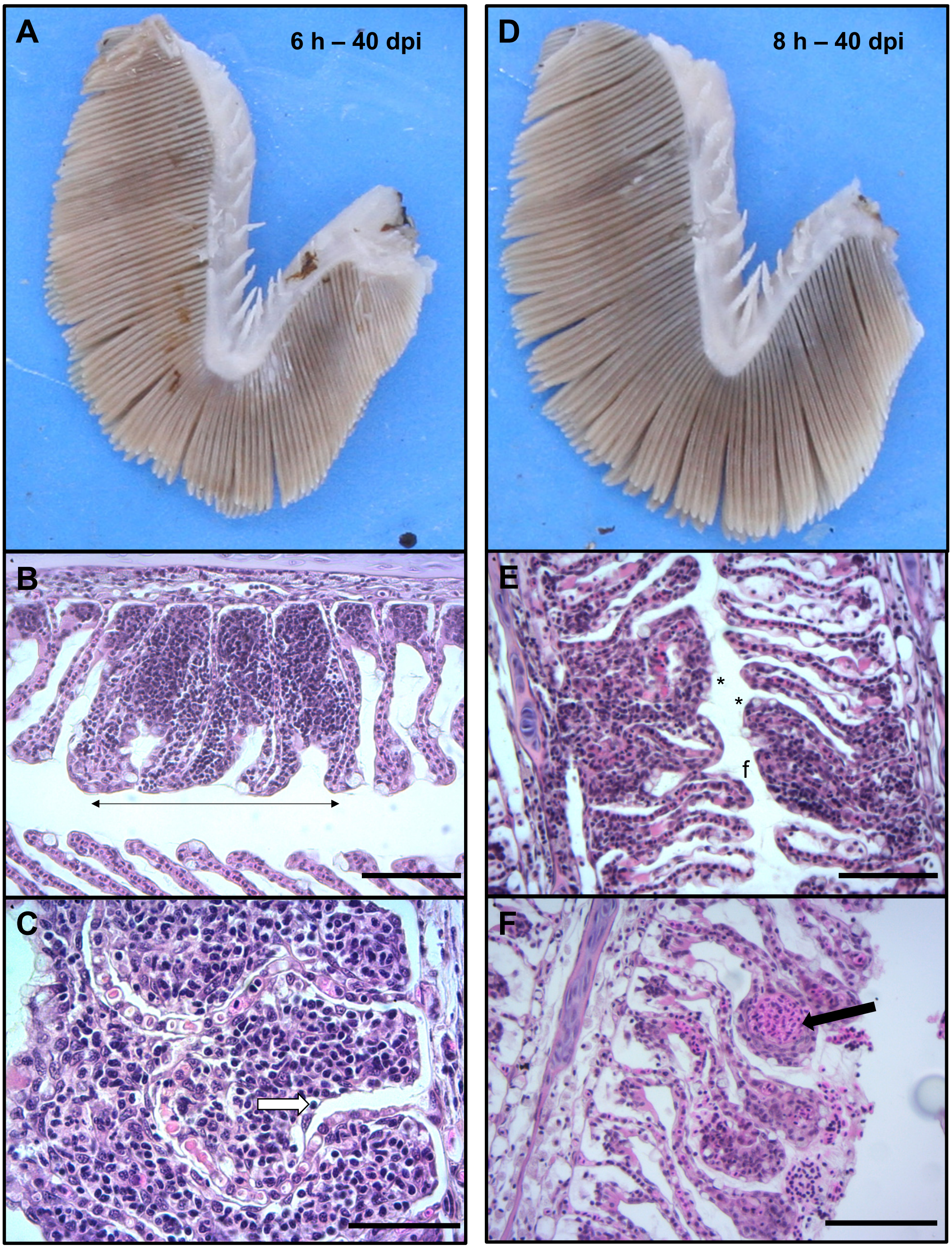

Figure S2. Gross images representative of 6 h (**A**) and 8 h (**D**) bathwater challenged fish (at 40 dpi) with no apparent AGD expression. Subsequent histological sections targeted areas of non-normal branchial pathology that was not associated to AGD pathology or disease expression; (**B**) – Nodules of predominately lymphocytic infiltration affecting several interlamellar units (double arrow) at 200x magnification, scale bar = 100µm. (**C**) – further magnification of lymphocytic nodule (400x mag) with obvious lymphocytes present (white arrow), scale bar = 50 µm. (**E**) – Area of congestion (200x mag) characterised by lamellar fusion (f) and epithelial lifting. Proliferative margins have concentrations of mucosal cells (*), scale bar = 100µm. (**F**) – Several telangiectasis lesions formed at the lamellar tips, with some evidence of lamellar thrombosis (black arrow) with fibrinous material, indicating lesion area was resolving. Scale bar = 50µm.
